# Patterns of mortality after prolonged follow-up of a randomised controlled trial using granulocyte colony-stimulating factor to maintain chemotherapy dose intensity in non-Hodgkin's lymphoma

**DOI:** 10.1038/sj.bjc.6604468

**Published:** 2008-07-01

**Authors:** A R Clamp, W D J Ryder, S Bhattacharya, R Pettengell, J A Radford

**Affiliations:** 1 Department of Medical Oncology, Cancer Research UK, University of Manchester, Christie Hospital, Wilmslow Rd., Manchester M20 4BX, UK; 2 Department of Haematology, St George′s Hospital Medical School, Cranmer Terrace, London SW17 ORE, UK

**Keywords:** non-Hodgkin's lymphoma, granulocyte colony-stimulating factor, mortality pattern, dose intensity, pharmacovigilance, second malignancy

## Abstract

The effect of utilising granulocyte colony-stimulating factor (G-CSF) to maintain chemotherapy dose intensity in non-Hodgkin's lymphoma (NHL) on long-term mortality patterns has not been formally evaluated. We analysed prolonged follow-up data from the first randomised controlled trial investigating this approach. Data on 10-year overall survival (OS), progression-free survival (PFS), freedom from progression (FFP) and incidence of second malignancies were collected for 80 patients with aggressive subtypes of NHL, who had been randomised to receive either VAPEC-B chemotherapy or VAPEC-B+G-CSF. Median follow-up was 15.7 years for surviving patients. No significant differences were found in PFS or OS. However, 10-year FFP was better in the G-CSF arm (68 *vs* 47%, *P*=0.037). Eleven deaths from causes unrelated to NHL or its treatment occurred in the G-CSF arm compared to five in controls. More deaths occurred from second malignancies (4 *vs* 2) and cardiovascular causes (5 *vs* 0) in the G-CSF arm. Although this pharmacovigilance study has insufficient statistical power to draw conclusions and is limited by the lack of data on smoking history and other cardiovascular risk factors, these unique long-term outcome data generate hypotheses that warrant further investigation.

One of the major dose-limiting toxicities of cytotoxic chemotherapy for non-Hodgkin's lymphoma (NHL) is myelosuppression and subsequent susceptibility to infection. Haemopoietic colony-stimulating factors (G-CSF and GM-CSF) are now commonly administered as an adjunct to cytotoxic chemotherapy in patients with NHL, to reduce the incidence of these toxicities based on data accrued from several randomised controlled studies (Pettengell *et al*, 1992; Bastion *et al*, 1993; Gerhartz *et al*, 1993; Aviles *et al*, 1994; Engelhard *et al*, 1994; Gisselbrecht *et al*, 1997; Zinzani *et al*, 1997; Doorduijn *et al*, 2003; Osby *et al*, 2003). It has also been suggested that CSF administration may improve overall survival and cure rate in this population by both decreasing treatment-related morbidity and, maintaining dose intensity, in accordance with the Goldie and Coldman (1983) hypothesis.

Although a recent meta-analysis of the above studies and those conducted in Hodgkin's lymphoma (Bohlius *et al*, 2004) has confirmed a 26% (95% CI 11–38%) reduction in the risk of febrile neutropenia with CSF prophylaxis, no overall survival benefit was noted despite a modest increase in the median cytotoxic dose intensities achieved in the arms receiving CSFs being reported in most studies.

However, the median follow-up of surviving patients in the trials included in the meta-analysis was relatively short at 4.4 years (range, 1.3–7.9 years) and all cause mortality was analysed. We therefore decided to analyse long-term follow-up data from the first published randomised study of G-CSF administration in NHL patients (Pettengell *et al*, 1992) to determine whether the use of G-CSF and consequent increase in chemotherapy dose intensity will impact long-term survival, overall morbidity or the development of unexpected late complications.

## Patients and methods

### Patients

Eighty patients aged 16–71 years with high-grade NHL (Kiel) of any disease stage/performance status commencing VAPEC-B chemotherapy were entered between August 1989 and March 1991. Forty-one were randomised to receive G-CSF (filgrastim (Amgen, Thousand Oaks, CA, USA)-230 mcg m^−2^ by daily subcutaneous injection for 13 weeks apart from days preceding and during myelosuppressive chemotherapy administration) in addition to chemotherapy and 39 received chemotherapy alone. Pretreatment characteristics of the two groups were well matched for prognostic factors (see Table 1). Thirty-four patients (83%) receiving G-CSF and 29 (74%) in the control group completed chemotherapy.

Patients randomised to receive G-CSF achieved 12% greater median dose intensity than control patients (95 *vs* 83%) – see Table 1 for median dose intensities of adriamycin, cyclophosphamide and etoposide.

Following completion of treatment, clinical details and disease status were regularly updated in the trial database either from out patient clinic annotations or by liaison with the patient's primary care physicians. Causes of death were retrieved from case notes or death certification. The case notes of all patients who had died since the previous publication or who had been designated as a non-lymphoma death in the trial database were reviewed by two authors (AC, JR).

### Statistical methods

The objectives of the study were to evaluate the long-term effects of the use of G-CSF and increased dose-intensity chemotherapy on overall survival and mortality patterns. Definitions of reported end points are as follows (Cheson *et al*, 2007).

Overall survival (OS) – time from randomisation to death from any cause.

Freedom from progression (FFP) – time from randomisation to first documentation of disease progression.

Progression-free survival (PFS) – time from randomisation to the first of disease progression or death from any cause.

All analyses were performed on an intention-to-treat basis. Kaplan and Meier, (1958) survival curves were drawn for overall survival and progression-free survival and compared between the treatment groups using the log-rank test. The proportions of patients having experienced an event at 10 years were estimated from the survival curves.

To assess the effects of specific causes of death, cumulative incidence curves were constructed in a competing risks framework. The cumulative incidence of death from cause x at time t is the probability of dying from x by time t in an environment where other causes of death are acting. The treatment groups were then compared using Gray's test (Gray, 1988).

A further analysis was also performed by fitting a multi-state Cox illness/death model (Andersen *et al*, 1993) for the three outcomes initial progression, death without progression and death following progression. This formulation of the Cox regression model allowed us to control for the potential effects of age, sex and international prognostic index score (International Non-Hodgkin's lymphoma prognostic factors project, 1993).

Relative mortality models (Andersen *et al*, 1993) were estimated for each trial arm separately using England and Wales death rates broken down by sex, age (integer years) and period (annually up until 2005, 2005 figures used for follow-up in 2006) obtained from the Human Mortality database (2008).

## Results

Forty-eight of the eighty initial patients had died at the time of this analysis. The median follow up of the 36 surviving patients was 15.7 years (range, 8.4–16.9 years). Of the 48 dead, 24 died in the G-CSF arm and 24 in the control arm. Ten-year overall survival figures were 51 and 46% in the G-CSF and control arms respectively and 10-year PFS 49 and 44%.

**Figure 1:**
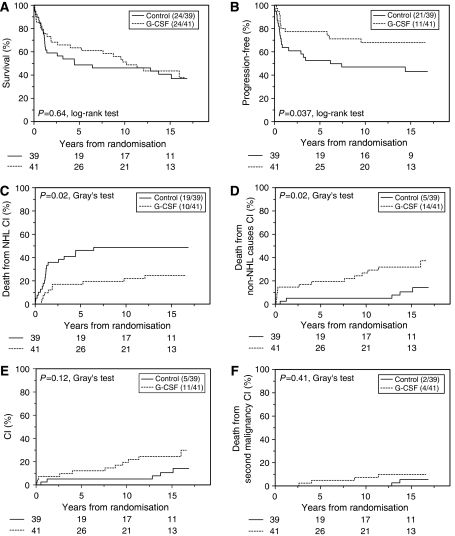
Survival end points. Survival curves comparing chemotherapy-alone arm (solid line) and chemotherapy+G-CSF arm (dashed line). Kaplan–Meier plots for (**A**) overall survival (**B**) freedom from progression. Cumulative incidence curves for (**C**) NHL-specific death (**D**) non-NHL deaths (**E**) death from causes other than progressive NHL and acute treatment-related infections (**F**) Deaths from second malignancy.

The Kaplan–Meier survival curves for OS and FFP are illustrated in Figure 1A and 1B respectively. Although OS and PFS (data not shown) were virtually identical in both arms, FFP was significantly higher in the interventional arm receiving G-CSF and consequently 12% higher dose intensity of chemotherapy (10-year FFP 68 and 47% for G-CSF and control arms respectively) suggesting an imbalance in the causes of death between the two arms. It was therefore decided to examine this in more detail and these results are summarised in Table 2.

Eleven of 41 patients in the G-CSF arm had documented progressive disease and 10 of these have subsequently died from NHL. In the control arm, 21 of the 39 patients had disease progression with 19 dying because of this (*P*=0.02). The cumulative incidence curves for lymphoma-specific death are shown in Figure 1C. Paradoxically, 14 patients in the G-CSF arm died from causes other than progressive NHL compared with five in the control arm (*P*=0.02) (Figure 1D).

To assess whether known lymphoma-related prognostic factors may have influenced these findings, we controlled these using a multi-state formulation of Cox regression model analysis (Table 3). Age, sex, international prognostic index score and treatment arm were analysed as separate variables. Unfortunately, baseline data on known cardiovascular and second cancer risk factors for example, smoking history, hypertension, serum cholesterol were not routinely collected and so could not be included in this analysis. However, the random allocation of patients to the two treatment arms should allow for the equal distribution of these risk factors between the two arms. This analysis confirmed that patients receiving G-CSF were less likely to experience lymphoma progression (HR 0.40; 95% CI 0.18–0.87) than controls but were more likely to die prior to lymphoma progression (HR 3.08; 95% CI 1.05–8.99).

Of the 14 non-NHL deaths in the G-CSF arm, three patients died from treatment-related infections, two from neutropenic infections during the 11-week treatment schedule and one from autopsy-confirmed invasive aspergillosis 3 months after commencement of chemotherapy. No patient in the control arm died from treatment-related infection.

Eleven deaths in the G-CSF arm and five in the control arms were not due to NHL or treatment-related infection (*P*=0.12) (cumulative incidence curves Figure 1E). It was impossible to assign a cause in two patients (one in each arm). Four patients who received G-CSF died from second malignancies compared to two in the control arm (Figure 1F). In total, second cancers were documented in seven patients, five in the G-CSF arm and two in the control arm. There were two cases of lung cancer (diagnosed 42 and 105 months after commencement of chemotherapy) and one each of acute myeloid leukaemia (28 months), breast cancer (76 months) and disseminated cancer (131 months- no histological diagnosis possible) in the G-CSF arm. In the control arm, two cases of lung cancer (54 and 159 months) were diagnosed. One case of localised cutaneous squamous cell carcinoma was also documented in the control arm.

Of the six other deaths in the G-CSF arm, five died from cardiovascular disease (three myocardial infarctions, one congestive cardiac failure and one ischaemic heart disease) and one from an intracerebral haemorrhage. In the control arm, one patient died from Pneumocystis carinii pneumonia 14 months after chemotherapy with no evidence of recurrent NHL and one from cirrhosis secondary to chronic hepatitis C infection. The causes of non-lymphoma deaths are summarised in Table 4.

**Figure 2:**
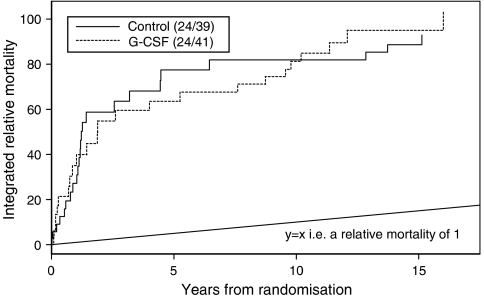
Relative mortality models. Relative mortality functions were estimated for each trial arm separately. While relative mortality rates in the chemotherapy alone arm return close to the underlying population with continued follow-up (slope gradient close to 1), rates appear to remain somewhat higher in the G-CSF treated arm.

One possible explanation for these findings is that because patients who were exposed to G-CSF appeared less likely to experience progression of lymphoma, they were exposed to other competing causes of death for longer than patients who did not receive G-CSF but with the proportional rate of non-lymphoma-related mortality being the same in both groups. To assess this possibility, we constructed relative mortality models for each trial arm separately using England and Wales death rates broken down by sex, age (integer years) and period (annually up until 2005, 2005 figures used for follow-up in 2006) obtained from the Human Mortality Database (Figure 2). This analysis tentatively indicates that after the period of early excess relative mortality associated primarily with lymphoma-related events, the relative mortality rate in the surviving subjects appears somewhat higher in the G-CSF treated subjects than in those who received chemotherapy alone.

## Discussion

There have been numerous randomised studies on the use of G-CSF during induction chemotherapy for high-grade NHLs (Pettengell *et al*, 1992; Bastion *et al*, 1993; Gerhartz *et al*, 1993; Aviles *et al*, 1994; Engelhard *et al*, 1994; Gisselbrecht *et al*, 1997; Zinzani *et al*, 1997; Doorduijn *et al*, 2003; Osby *et al*, 2003). While a recent meta-analysis (Bohlius *et al*, 2004) of these indicates a clinical benefit in terms of reducing the risk of febrile neutropenia, no clear improvement was demonstrable in terms of tumour response or survival parameters. However, follow-up was short (median 4.4 years) and cause-specific survival was not analysed.

In this report, we detail long-term pharmacovigilance follow up of the first randomised study of G-CSF during induction chemotherapy for high-grade NHL (Pettengell *et al*, 1992). No such extended follow-up has previously been presented. While progression-free and overall survival figures were similar in the cohort of patients receiving G-CSF and those in the control arm (Table 1), a difference was noted in the percentage of patients free from lymphoma progression at 10 years (68% G-CSF intervention arm *vs* 47% control arm). This prompted us to further investigate the mortality patterns of the two study groups, which were well-balanced at original randomisation for age, sex and performance status (Table 1). Nineteen patients in the control group died from progressive NHL compared with 10 in the G-CSF group (*P*=0.02) while five died from other causes in the control group compared with 14 in the G-CSF group (*P*=0.02). Despite the small size and retrospective nature of our analysis, this observation could support the hypothesis that the use of G-CSF to facilitate chemotherapy administration allows greater lymphoma-specific survival at the expense of death from other causes. However, our analysis is limited by the lack of baseline information on major risk factors for both second malignancies and cardiovascular disease including smoking and family histories, hypertension, diabetes and cholesterol levels. The random allocation of patients to treatment arm will have limited uneven distribution of these variables between the two treatment arms but we cannot exclude biased distribution as an explanation of our findings.

An excess of deaths from acute treatment-related infective complications were noted in the intervention arm of our study. The reasons for this are unclear. Identical cytotoxic dose modification criteria were applied in both groups and G-CSF administration reduced the risk of febrile neutropenia. No increased on-chemotherapy mortality was seen associated with G-CSF administration in the Bohlius meta-analysis (relative risk– 0.93 95% CI 0.60–1.43) (Bohlius *et al*, 2004).

Eleven patients died from other causes in the G-CSF arm compared with five in the control arm (*P*=0.12). The occurrence of deaths from second malignancy and cardiovascular disease which account for the majority of these (9; G-CSF *vs* 2; control) are of particular interest as it is now apparent from large epidemiologic analyses of NHL databases that long-term NHL survivors are at increased risk from these treatment-related complications.

### Second malignancy

An increased incidence of second malignancy has been reported in most but not all retrospective cohort studies (Travis *et al*, 1993; André *et al*, 2004; Moser *et al*, 2006a; Mudie *et al*, 2006). Although André *et al* (2004) failed to demonstrate an overall increased risk of second cancer after a median follow-up of 74 months in 2837 patients with NHL treated with adriamycin-cyclophosphamide-vindesine–bleomycin–prednisolone, they noted an excess of myeloid malignancies and an increased risk of lung cancer in males. Other studies have demonstrated a 20–30% overall increased incidence of malignancy primarily due to increased rates of leukaemia, lung and possibly colorectal cancers (Travis *et al*, 1993; Moser *et al*, 2006a; Mudie *et al*, 2006). It is also apparent that this elevated risk persists for at least 15 years after NHL treatment and is inversely related to age at diagnosis.

There are several studies that suggest that the use of G-CSF may increase the risk of secondary myeloid malignancies during follow-up (Relling *et al*, 2003; Hershman *et al*, 2007) however, evidence from NHL studies are lacking. G-CSF to facilitate dose dense chemotherapy (two *vs* three weekly CHOP) in the first-line therapy of aggressive NHL (Pfreundschuh *et al*, 2004a, 2004b) improved 5-year event-free and overall survivals without any significant increase in the incidence of second cancers in the dose dense treatment arms after 58 months median follow-up. The total dose of chemotherapy delivered was identical in both arms and it is possible that follow-up in these studies is not yet long enough to detect any differences.

Although increased dose intensity may be postulated as one contributory factor to an increase in the incidence of second malignancies in patients exposed to G-CSF in this study, experimental studies have implicated a mechanistic role for CSFs in the potentiation of neoplastic progression. CSFs have been implicated as direct promoters of both tumour cell growth and migration (Mueller *et al*, 1999; Obermuller *et al*, 2004) and have also been shown to have paracrine as well as autocrine tumour promoting properties as they modulate the tumour stroma and enhance angiogenesis in a squamous skin carcinoma model (Obermuller *et al*, 2004).

### Cardiovascular disease

Although the long-term risk of cardiovascular disease in patients treated for aggressive NHL has not been extensively investigated, an increased incidence of chronic heart failure (SIR 5.4, 95% CIs 4.1–6.9) has been reported in one retrospective cohort study of 592 patients (Moser *et al*, 2006b). Although no overall increase in myocardial infarction was noted, the risk was elevated in patients with at least 5 years follow-up. This is consistent with data from the Collaborative British Cohort Study in Hodgkin's disease (Swerdlow *et al*, 2007), which noted increased risks of myocardial infarction associated with anthracycline and vincristine-containing chemotherapy regimens that were significant even in the absence of supradiaphragmatic radiotherapy.

How G-CSF could affect the long-term incidence of cardiovascular disease is less clear. Although patients receiving G-CSF achieved a 12% increased adriamycin dose intensity, total dose (200 mg m^−2^) was still well below the conventional ceiling dose for clinically apparent cardiotoxicity of 400–450 mg m^−2^. It is also intriguing to note that G-CSF appears to protect from anthracycline cardiomyopathy in animal models (Hou *et al*, 2006) and has been proposed as a possible preventive agent in the clinic (Takemura and Fujiwara, 2007).

To conclude, this unique long-term follow-up data of the first randomised study investigating the use of G-CSF in the first-line chemotherapeutic treatment of high-grade NHL suggests that although overall survival is not altered by this intervention, the pattern of mortality may be changed. Although potential long-term increased risk of second malignancy and cardiovascular disease in patients receiving G-CSF is intriguing and may have significant implications, the small size of the original study cohorts, lack of information on other known risk factors and the *post hoc* nature of this analysis limits the conclusions that can be drawn. However, this analysis emphasises the need for long-term and detailed follow-up of patients enrolled into such studies and the sharing of these data for the purposes of meta-analysis.

## Figures and Tables

**Table 1:** Patient characteristics at trial entry and dose intensity of treatment received

	**G-CSF (*n*=41)**	**Control (*n*=39)**
*Age (years)*		
Median (range)	51 (16–67)	53 (22–71)
		
*Gender*		
Male	27	26
Female	14	13
		
*ECOG PS*		
0	6	4
1	21	19
2	11	13
3	3	3
		
*Disease stage*		
I	9	8
II	12	14
III	5	3
IV	15	14
		
B symptoms	15	17
		
*Histology (working formulation)*		
High	35	33
Intermediate	5	5
Unclassified	1	1
		
*IPI risk grouping*		
Low	25	26
Low-intermediate	5	6
High-intermediate	6	2
High	5	5
		
*Median dose intensity*		
Whole regimen	95%	83%
Adriamycin	96%	85%
Cyclophosphamide	96%	83%
Etoposide	94%	82%

ECOG PS=Eastern Cooperative Oncology Group performance status.

**Table 2:** Patient status at last follow-up

	**Patients treated**	**Alive and well (%)**	**Alive with progressive disease (%)**	**Dead from NHL (%)**	**Dead from treatment-related infection (%)**	**Dead from second malignancy (%)**	**Dead from other causes (%)**	**Cause of death not identified (%)**
G-CSF	41	17 (41)	0	10 (24)	3 (7)	4 (10)	6 (15)	1 (2)
Control	39	13 (33)	2 (5)	19 (46)	0	2 (5)	2 (5)	1 (3)

Median follow-up for surviving patients is 15.7 years (range, 8.4–16.9).

**Table 3:** Hazard ratio estimates (95% confidence interval) for three transitions in a multi-state cox illness/death model

	**Progression (*n*=32/80)**	**Death without progression (*n*=18/80)**	**Death following progression (*n*=30/32)**
Male vs female	1.07 (0.50, 2.29) *P*=0.86	1.14 (0.41, 3.16) *P*=0.80	0.99 (0.36, 2.71) *P*=0.98
Age (years)	0.99 (0.96, 1.02) *P*=0.51	**1.13** (**1.05, 1.21) *P*=0.002**	1.02 (0.98, 1.06) *P*=0.34
IPI (integer score)	**1.57** (**1.23, 2.01) *P*=0.0003**	1.19 (0.84, 1.69) *P*=0.34	1.38 (0.98, 1.94) *P*=0.06
G-CSF vs control	**0.40** (**0.18, 0.87) *P*=0.02**	**3.08** (**1.05, 8.99) *P*=0.04**	0.69 (0.25, 1.85) *P*=0.46

IPI=international prognostic index.

Significant hazard ratios are shown in bold.

**Table 4:** Causes of non-lymphoma deaths

**G-CSF**		**Control**	
**Cause**	**TTD (mos)**	**Cause**	**TTD (mos)**
*Second cancer*			
AML	28	NSCLC	154
NSCLC	48	NSCLC	165
NSCLC	104		
Metastatic carcinoma	131		
			
*Cardiovascular disease*			
MI	2		
MI	3.5		
Cardiac failure	115		
MI	134		
IHD	192		
			
*Other*			
Intracerebral haemorrhage	3	Not known	6.5
Not known	92	PCP	14
		Cirrhosis secondary to Hepatitis C	182

AML=acute myeloid leukaemia; IHD=ischaemic heart disease; MI=myocardial infarction; NSCLC=Non-small cell lung cancer; PCP=pneumocystis carinii pneumonia.

Deaths from treatment-related infections are not included.
